# Retracted: Vesicle-Associated Membrane Protein-Associated Protein A Is Involved in Androgen Receptor Trafficking in Mouse Sertoli Cells

**DOI:** 10.1155/2019/7683504

**Published:** 2019-09-08

**Authors:** International Journal of Endocrinology

*International Journal of Endocrinology* has retracted the article titled “Vesicle-Associated Membrane Protein-Associated Protein A Is Involved in Androgen Receptor Trafficking in Mouse Sertoli Cells” [1]. A reader noted that the article is one of the series of four articles on androgen receptor (AR) localization/trafficking in a mouse Sertoli cell line published by the authors in 2017-18, two in the Hindawi journal *International Journal of Endocrinology* and two in *Cellular Physiology and Biochemistry*. The details of the articles are as follows:

1. Q. Deng, Y. Wu, Z. Zhang, et al., “Androgen receptor localizes to plasma membrane by binding to Caveolin-1 in mouse sertoli cells,” *International Journal of Endocrinology*, vol. 2017, Article ID 3985916, 8 pages, 2017. https://doi.org/10.1155/2017/3985916. Published 31 May 2017, on AR localization to the membrane depending on caveolin [2].
2. Q. Deng, Z. Zhang, Y. Wu, et al., “Non-genomic action of androgens is mediated by rapid phosphorylation and regulation of androgen receptor trafficking,” *Cellular Physiology and Biochemistry*, vol. 43, pp. 223–236, 2017. https://doi.org/10.1159/000480343. Published 29 August 2017, on phosphorylation when AR localizes under testosterone treatment. Figure 1 is the same as Figure 1 in IJE/3985916. This does not cite IJE/3985916 [3].
3. The retracted article. Published 4 March 2018, on the role of VAPA (a vesicle-associated protein) and OSBP in AR trafficking. In Figure 1(c), the top-left panels are the same as Figure 2(a) in IJE/3985916, though the western blots are intended to represent AR, VAPA, and pan cadherin versus AR, caveolin, and E-cadherin, respectively. This cites IJE/3985916 and CPB/480343 [1].
4. Q. Deng, C. He, Y. Wu, et al., “CSN6 and Rab34 are involved in androgen receptor trafficking in mouse testicular sertoli cells,” *Cellular Physiology and Biochemistry*, vol. 47, pp. 2360–2368, 2018. https://doi.org/10.1159/000491608. Published 9 July 2018, on the role of CSN6 and Rab34 in AR trafficking. Figure 1 is the same as Figures 1(a) and 1(b) in IJE/4537214 and the text in the introduction and the beginning of the discussion is similar to IJE/4537214. This cites IJE/3985916 and IJE/4537214 [4].

The negative controls of AR expression in IJE/3985916 (Figure 3(c)) and IJE/4537214 (Figure 2(b)) with or without testosterone look similar, but they are different from CPB/491608 (Figures 2(c) and 4(c)); in the former two articles, the band without testosterone is not so faint as in the latter article.

The authors apologized and said the articles were all prepared at around the same time, and for articles IJE/3985916 and CPB/480343, the data in Figure 1 in both of the articles are critical for the subsequent experiments. They explained that, in articles IJE/3985916 and IJE/4537214, they conducted two experiments at the same time, the mass spectrometry (MS) and the protein phosphorylation signaling pathway array, and that several candidate proteins involved in AR trafficking were screened by MS and they tried to verify the data by immunoprecipitation.

Additionally, the authors clarified that they conducted each of the experiments at least three times. For the western blot assay, they kept the amount of protein and exposure time the same and clarified that the cell response to testosterone may be a little different though the trend was the same. They added that it might also be due to the negative control provided by the company indicated in the article.

The authors provided duplicate figures, but the editorial board advised to retract the article.
